# Non-Destructive Evaluation of Depth of Surface Cracks Using Ultrasonic Frequency Analysis

**DOI:** 10.3390/s140917146

**Published:** 2014-09-15

**Authors:** Shiuh-Chuan Her, Sheng-Tung Lin

**Affiliations:** Department of Mechanical Engineering, Yuan Ze University, Chung-Li 320, Taiwan; E-Mail: s852723@mail.yzu.edu.tw

**Keywords:** surface crack, non-destructive evaluation, ultrasonic, Fourier transform

## Abstract

Ultrasonic is one of the most common uses of a non-destructive evaluation method for crack detection and characterization. The effectiveness of the acoustic-ultrasound Structural Health Monitoring (SHM) technique for the determination of the depth of the surface crack was presented. A method for ultrasonic sizing of surface cracks combined with the time domain and frequency spectrum was adopted. The ultrasonic frequency spectrum was obtained by Fourier transform technique. A series of test specimens with various depths of surface crack ranging from 1 mm to 8 mm was fabricated. The depth of the surface crack was evaluated using the pulse-echo technique. In this work, three different longitudinal waves with frequencies of 2.25 MHz, 5 MHz and 10 MHz were employed to investigate the effect of frequency on the sizing detection of surface cracks. Reasonable accuracies were achieved with measurement errors less than 7%.

## 1. Introduction

The safety of civil structures such as bridges, dams, nuclear power plants, *etc.* directly affects the security of both the environment and human beings. It has long been recognized that there is a need to inspect structures around people in order to prevent failures. Structural health monitoring (SHM) is an important research topic and a challenging task bringing together non-destructive evaluation (NDE) and civil engineering communities [1]. The choice of the specific NDE method depends on many factors including the size, orientation and location of the flaw, as well as the type of material, *etc.* [2–4]. Researchers working in the field of SHM have proposed many techniques. Some of these techniques include Acoustic-Ultrasound [5,6], electrochemical sensors [7], and Fiber Bragg grating sensors [8,9]. Among them, ultrasonic has been extensively employed in nondestructive evaluation, and it is one of the few NDE techniques suitable for use in a structure-integrated damage monitoring system [10,11]. Monitoring the onset and growth of cracks in critical structures has been a research area of great interest in the field of structural health monitoring. Mi *et al.* [11] developed an ultrasonics-based SHM technique for detecting initiation and growth of cracks emerging from rivet holes during fatigue loading. Recently, work has focused on the development of ultrasonic methods for *in situ* monitoring of structural health. Some examples include using surface acoustic wave modulation to monitor growing cracks [12], and vibration modal analysis for characterizing fatigue cracks [13] and Lamb waves for detecting cracks in plate structures [14]. Ultrasound has considered to be a suitable technique for characterizing surface crack, in particular through the use of Rayleigh wave and acoustic emission. Rayleigh waves are elastic waves confined to a thin layer near the free surface. It can propagate over curved surfaces with little distortion provided that the curvature of the surface is greater than the wavelength of the Rayleigh wave. This allows for long range inspection of structures with wave propagation distances of several meters. However, this method can be limited by the presence of multiple wave-modes, and resolution can be limited if defects are located close to one another or have insufficient depth to cause a significant reflection of the incident wave [15,16]. Another useful property of the Rayleigh wave is that the speed of propagation is independent of the frequency in isotropic materials [17]. Acoustic emission technique has been widely used in integrity testing and evaluation for materials. It often needs very high pressure to produce enough stress in the specific areas. The external mechanical loading might cause additional damage [18]. Electromagnetic acoustic emission (EMAE) uses electromagnetic stimulation to produce acoustic emission (AE) signals, can be employed to detect and locate the small hidden cracks. Electromagnetic stimulation can be loaded according to the actual demands so that it can reduce the time required of traditional AE load (needs a long-term load time) [18]. EMAE provides a number of advantages over the conventional generation by piezoelectric transducers, namely high spatial resolution, non-contact generation and detection of ultrasonic waves, and ability to operate on curved and rough surfaces [19].

Electromagnetic method (EM) is an alternative method used within the field of nondestructive evaluation in recent years. All electromagnetic methods are basing on the electromagnetic principle and involving the Maxwell's equations. A variety of electromagnetic methods, e.g., eddy current, microwave, magnetic flux leakage, has been proposed to detect the anomalies or defects [20]. Among them, eddy current is often employed to detect superficial or sub-surface defects in metallic components. In contrast to conventional eddy current technique, where the excitation is limited to single frequency, pulsed eddy current (PEC) technique takes advantage of the broad frequency spectrum induced by a pulse excitation in time domain. The broad band frequency has been shown to be particularly useful for detecting deeply hidden sub-surface defects [21,22]. Response signals of PEC provide good information in describing the conditions of interior structures [23]. The main advantage of pulsed eddy current is the ability to penetrate deeply in the conductive specimen. In addition, the simplicity of the technique and the ease with which useful information can be obtained from the time domain signals. On the other hand, the payback is the lack of phase information, which is crucial in applications with harmonic excitations, where the phase difference is used for canceling or discriminating against unwanted signals such as the always present lift-off [24]. Edwards *et al.* [25] proposed a dual EMAT and PEC non-contact probe combining two techniques, which are sensitive to surface and subsurface defects, in a single probe: pulsed eddy current and two electro-magnetic acoustic transducers (EMATs). By integrating these two techniques, they were able to accurately characterize surface breaking defects with depths of up to 20 mm. The dual-probe approach can bring many benefits, but most importantly a higher accuracy for sizing and detection of defects, with a reduced time and cost as compared to using separate NDT devices [25]. In comparison with ultrasonic technique, EM is restricted to conducting and dielectric materials and the instrumentation required in EM is more complicated.

Engineering components subjected to external loading can develop cracks. The detailed analysis of failure mechanisms has shown that the fatigue lifetime of several structural components is considerably influenced by the growth rate of short cracks [26]. Sizing of short cracks using nondestructive testing methods is therefore essential. It allows the crack growth to be monitored and define inspection intervals for safety relevant components [27]. One widely used nondestructive evaluation method for crack detection and characterization is ultrasonic testing [28]. Baby *et al.* [29] measured the size of surface break crack using the time-of-flight diffraction (TOFD) technique with a mean error of 0.13 mm. Kimoto *et al.* [30] evaluated the depth of surface cracks using the anti-plane shear wave (SH wave). The ultrasonic testing is based on the detection and the interpretation of the ultrasonic waves reflected by cracks. Various techniques of signal processing like the wavelet transform [31], split spectrum processing [32] and Hilbert transform [33] were introduced. In this study, the size of surface crack was detected using longitudinal waves. The time and frequency analyses were employed to increase the detection and improve the localization of these defects. A signal processing technique basing on the Fourier transform is presented for significantly improving the accuracy of the ultrasonic testing.

## 2. Depth of Surface Crack

**Figure 1.**
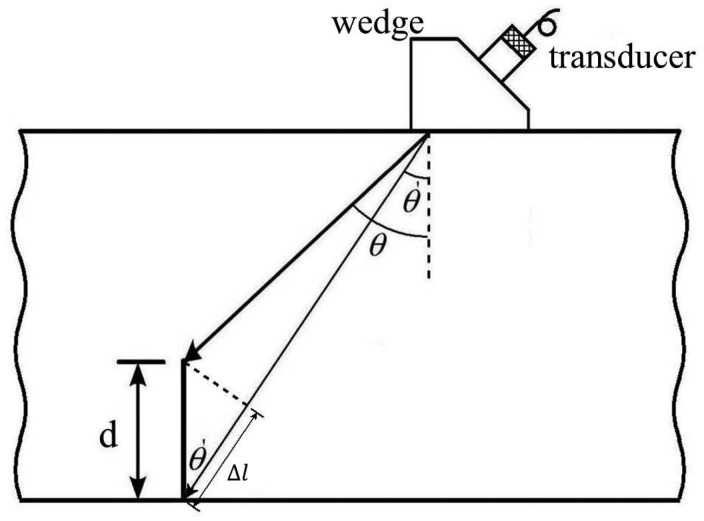
Experimental setup of the ultrasonic testing.

**Figure 2.**
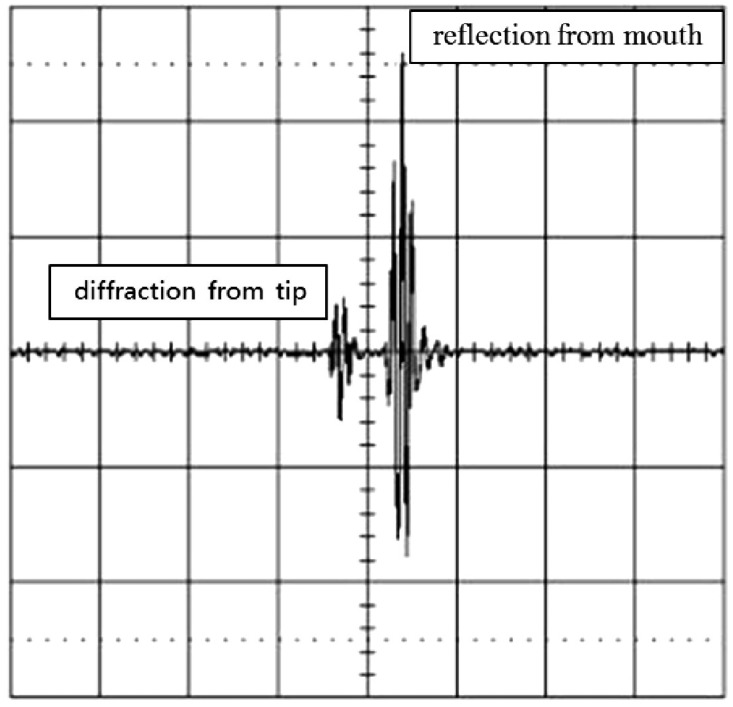
Reflection waves from the surface crack.

The experimental setup of the ultrasonic testing is shown in Figure 1. An ultrasonic transducer was mounted on a wedge with an angle of *θ* and placed on the top of the test specimen. The wave enters the wedge to impose a desired angle on the ultrasonic beam, and from the wedge transmits to the specimen. When an ultrasonic wave impinges on a surface crack in a solid at an incident angle of *θ*, the emitted energy distribution will be the result of contribution from two components as shown in Figure 1. The first type of pulse component is the diffraction of a ray at the tip of the crack. The second type of pulse component is the wave reflected from the mouth of the surface crack. When an ultrasonic wave impinges on a crack, the sharp tip will diffract the incident ultrasonic wave, creating a spherical wave front from the mode conversion. The arrival of the spherical wave at the receiver can be used to locate the tip and measure the depth of the crack, although the signals involve the mode conversions over a portion of the path difference. In this work, the ultrasonic testing is operated in a pulse-echo mode. Typical waveforms recorded by an oscilloscope are shown in Figure 2, where the first pulse corresponds to the signal diffracted from the crack tip and the second pulse is the reflection wave from the crack mouth. The pulses are received by the same transducer as shown in Figure 1. The depth of the surface crack can be determined by measuring the difference of the arrival time between these two pulses.

The difference of the travelling paths between the wave diffracted at the tip and the wave reflected from the crack mouth shown in Figure 1 can be calculated as

(1) $$\Delta l=\frac{C_{s}\Delta t}{2}$$

where *C_s_* denote the velocity of the incident wave; *Δt* is the difference of the arrival time between these two pulses; *Δl* is the path difference between the two pulses.

The depth of the surface crack is readily determined as follow,

(2) $$d=\frac{\Delta l}{\cos\theta^{\prime }}=\frac{C_{s}\Delta t}{2\cos\theta^{\prime }}$$

where *θ*′ is the angle between the incident wave and crack mouth as shown in Figure 1. For a short crack the angle *θ*′ is approximate to the incident angle *θ*′ of the incident wave. Thus, the depth of the surface crack can be rewritten as

(3) $$d=\frac{C_{s}\Delta t}{2\cos\theta }$$

The depth of the surface crack can be determined by substituting the incident angle *θ* and the difference of the arrival time Δ*t* between the wave diffracted at the tip and the wave reflected from the crack mouth into Equation (3). In this work, the time difference Δ*t* is evaluated using Fourier transform as described in the following section.

## 3. Fourier Transform

Fourier transform is one of the most widely used techniques in the signal processing. It converts signals from time domain to frequency domain. In this work, the ultrasonic waves reflected from both the tip and mouth of the surface crack are transformed to frequency domain through Fourier transformation. The time delay Δ*t* between the two reflection waves can be extracted from the frequency spectrum.

The Fourier transformation of a time domain signal *f*(*t*) is defined as

(4) $$I\left[f\left(t\right)\right]=F\left(\omega \right)={\left(2\pi \right)}^{\overset{-1}{\;}/\underset{2}{\;}}\int_{-\infty }^{\infty }f\left(t\right)e^{-i\omega t}dt$$

where *ω* denotes the angular frequency; 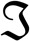 refers to the Fourier transform.

The magnitude of the Fourier transform is

(5) |I[f(t)]|=|F(ω)|

All signals encountered in practice will be of finite time duration, either inherently or because of the finite width of the electronic gate in typical frequency-analysis equipment [24]. Thus, Equation (4) can be rewritten as

(6) $$F\left(\omega \right)={\left(2\pi \right)}^{\overset{-1}{\;}/\underset{2}{\;}}\int_{-T}^{T}f\left(t\right)e^{-i\omega t}dt$$

where *f*(*t*) is centered about t = 0 and is of duration 2T.

If the Fourier transform of *f*(*t*) is *F*(*ω*), then the transform of *f*(*t* − *t*_0_) is

(7) $$I\left[f\left(t-t_{\circ }\right)\right]=e^{-i\omega t_{\circ }}F\left(\omega \right)$$

where *f*(*t* – *t_0_*) is the same as *f*(*t*) except that it is centered at *t* = *t_0_*.

**Figure 3.**
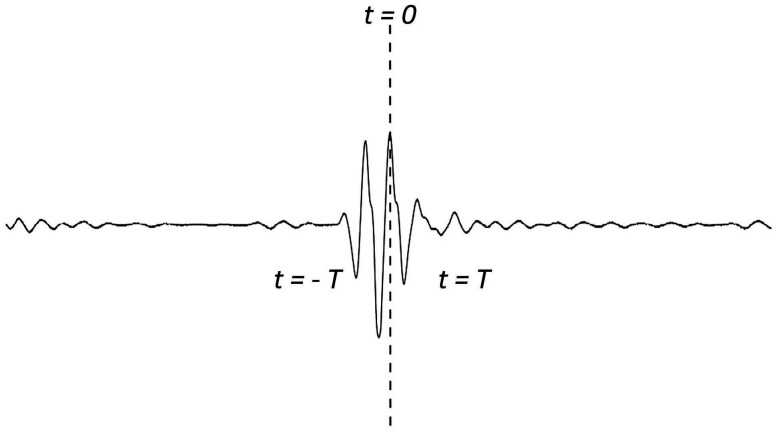
Ultrasonic pulse *f*(*t*) with duration of 2T.

**Figure 4.**
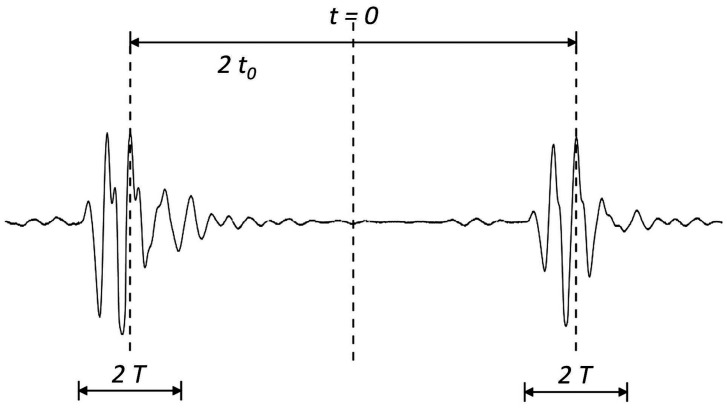
Two identical ultrasonic pulses with time delay of 2 *t*_0_.

Now consider the case of an ultrasonic pulse *f*(*t*) with a duration of 2T as shown in Figure 3. A second identical pulse is added and that the total delay between the two pulses is 2*t_0_* as shown in Figure 4. They are shown separated in time for clarity. It is worth to note that these two pulses may overlap. In this study, the two pulses represent the reflection waves from the tip and mouth of the surface crack as shown in Figure 2.

From Equations (5) and (7), the magnitude of the resultant Fourier transform is

(8) $$\left|I\left[f\left(t-t_{0}\right)\right]+I\left[f\left(t+t_{0}\right)\right]\right|=\left|e^{-i\omega t_{0}}F\left(\omega \right)+e^{i\omega t_{0}}F\left(\omega \right)\right|=\left|2\cos\omega t_{0}\right|\left|F(\omega )\right|$$

Comparing Equations (5) and (8), the magnitude of the resultant spectrum is that of the either pulse alone modulated by |2 cos *ω t*_0_|. The maxima and minima of the resultant spectrum are dependent on the time delay *t*_0_.

Approximating the minima of Equation (8) by the minima of |2 cos *ω t*_0_|, leads

(9) $$\omega t_{0}=\frac{2n+1}{2}\pi$$

where *n* is an integer.

Substituting *ω* = 2*πf* into Equation (9), yields

(10) $$f_{n}=\frac{1}{2t_{0}}\frac{2n+1}{2}n=0,1,2,3,‐‐‐‐$$

where *f_n_* are the frequencies of the minima.

The separation between minima is

(11) $$\Delta f=f_{n}-f_{n-1}=\frac{1}{2t_{0}}\left[\frac{2n-1}{2}-\frac{2(n-1)-1}{2}\right]=\frac{1}{2t_{0}}=\frac{1}{\Delta t}$$

Thus, the time delay Δ*t* between the two pulses can be determine as follow

(12) $$\Delta t=\frac{1}{\Delta f}$$

where Δ*f* is the separation between minima in the frequency spectrum.

A numerical example is presented to illustrate the feasibility of measuring the time delay between two pulses using Fourier transform.

### Numerical Example

A Gaussian function is adopted to simulate the ultrasonic wave

(13) $$f\left(t\right)=A\times e^{-{\left(\overset{t}{\;}/\underset{\sigma }{\;}\right)}^{2}}\times \sin\left(2\pi f_{\circ }t\right)$$

where A, *f*_0_ and *σ* are the amplitude, nominal frequency and decay constant, respectively.

Two pulses are generated as follows

(14) $$f_{1}\left(t\right)=0.5e^{-{\left[\overset{t}{\;}/\underset{2.4\times 10^{-7}}{\;}\right]}^{2}}\sin\left(2\pi \times 5\times 10^{6}t\right)$$

(15) $$f_{2}\left(t\right)=0.5e^{-{\left[\overset{t}{\;}/\underset{1.4\times 10^{-7}}{\;}\right]}^{2}}\sin\left(2\pi \times 5\times 10^{6}t\right)$$

**Figure 5.**
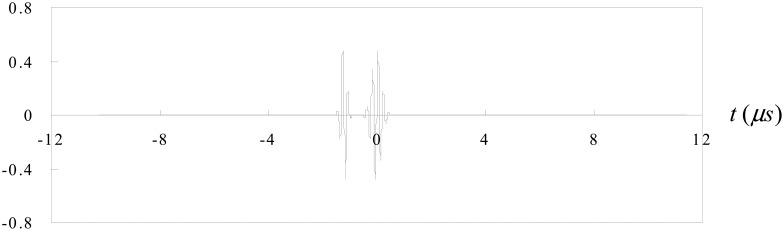
Two pulses with time delay Δ*t* = 1.2*μs*.

**Figure 6.**
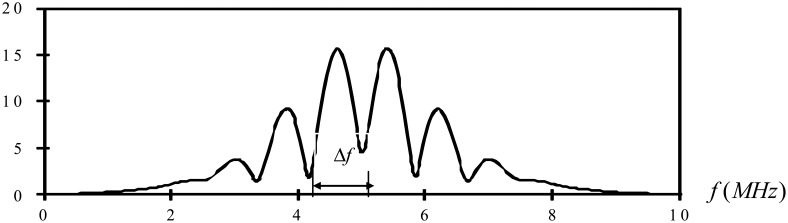
Fourier transform of two pulses with time delay Δ*t* = 1.2*μs*.

**Figure 7.**
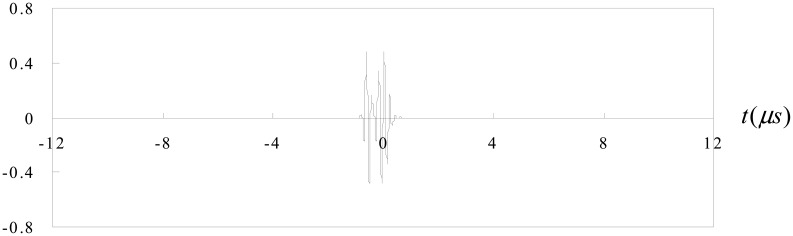
Two pulses with time delay Δ*t* = 1.55*μs*.

**Figure 8.**
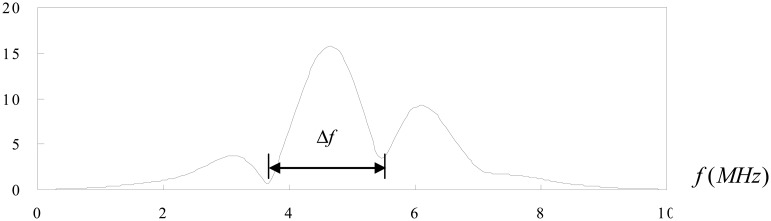
Fourier transform of two pulses with time delay Δ*t* = 0.55*μs*.

Case 1 The time delay between the two pulses is Δ*t* = 1.2*μs* as shown in Figure 5. The two pulses are well separated.
  Fourier transform for the two pulses is shown in Figure 6.
  The separation of minima in the frequency spectrum is Δ*f* = 0.83*MHz* as shown in Figure 6. Substituting Δ*f* into Equation (12), leads to the time delay between the two pulses Δ*t* = 1.204*μs*, which is in a close agreement with the exact time delay Δ*t* = 1.2*μs*.
Case 2 The time delay between the two pulses is Δ*t* = 1.55*μs* as shown in Figure 7. The two pulses are partial overlap.
  Fourier transform for the two pulses is shown in Figure 8.
  The separation of minima in the frequency spectrum is Δ*f* = 1.807*MHz* as shown in Figure 8. Substituting Δ*f* into Equation (12), leads to the time delay between the two pulses Δ*t* = 0.553*μs*, which is in a close agreement with the exact time delay Δ*t* = 0.55*μs*.

## 4. Experimental Test Results

**Figure 9.**
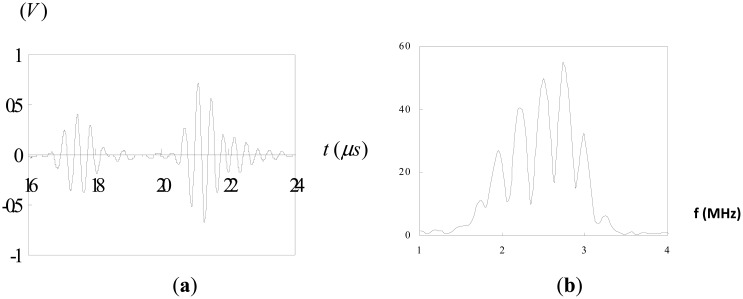
Reflection from a surface crack with 8 mm crack depth using 2.25 MHz longitudinal wave: (**a**) time domain; (**b**) frequency domain.

**Figure 10.**
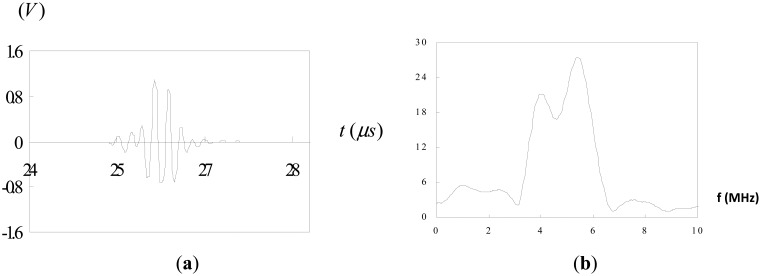
Reflection from a surface crack with 1 mm crack depth using 5 MHz longitudinal wave: (**a**) time domain; (**b**) frequency domain.

**Figure 11.**
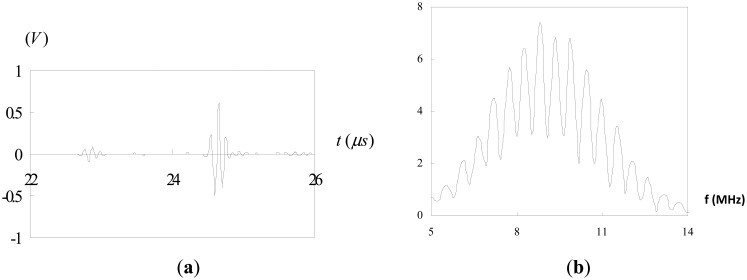
Reflection from a surface crack with 4 mm crack depth using 10 MHz longitudinal wave: (**a**) time domain; (**b**) frequency domain.

The test block is made of 304 steel with dimensions of 160 mm × 24 mm × 24 mm. The test block was spark-eroded by a wire with 0.3 mm diameter from the outer surface through the full thickness of the block to simulate a surface crack. A series of test block contained a surface-breaking crack with various crack depths ranging from 1 mm to 8 mm was fabricated. An ultrasonic transducer was mounted on a wedge with the angle of 45° as shown in Figure 1. The pulse-echo technique was employed to measure the depth of the surface crack. Three sets of measurement were taken, using 2.25 MHz, 5 MHz and 10 MHz longitudinal waves. The velocity of the longitudinal wave in 304 steel is 6.13 mm/ms. In order to carry out the Fourier transform, *f*(*t*) must satisfy the Dirichelt conditions. For any physically realizable signals, the Dirichelt conditions can be satisfied. Thus, it is justified with the mathematical applicability of the Fourier transform to the present problems. In this work, the fast Fourier transform (FFT) was conducted using the Matlab software, which is a well-known and widely used mathematical software. The Hanning window function is employed in the fast Fourier transform. Figure 9 shows the reflection from a surface crack with 8 mm crack depth using 2.25 MHz longitudinal wave. Figure 9a depicts the time domain signals where the first pulse represents the diffracted wave at the crack tip and the second pulse is the reflected wave from the crack mouth. The corresponding Fourier transform is plotted in Figure 9b. The separation of minima (Δ*f*) in frequency spectrum can be extracted from Figure 9b. Substituting Δ*f* into Equation (12) leads to the time delay Δ*t* between the diffraction wave and reflection wave. The depth of surface crack is readily determined by substituting the time delay Δ*t*, wave velocity *C_s_* = 6.13 mm/ms and incident angle *θ* = 45° into Equation (3). Figure 10 shows the reflection from a surface crack with 1 mm crack depth using 5 MHz longitudinal wave. Figure 11 shows the reflection from a surface crack with 4 mm crack depth using 10 MHz longitudinal wave. Table 1 lists the experimental results of the crack depth measured by the ultrasonic technique using 2.25 MHz, 5 MHz and 10 MHz longitudinal waves. Reasonable accuracies were achieved with measurement errors less than 7%. It appears that the capability of measuring the crack depth is dependent on the frequency of the incident wave. Ultrasonic wave with center frequency 2.25 MHz is not able to detect a surface crack with crack depth less than 3 mm, while the 10 MHz longitudinal wave cannot detect the crack depth longer than 6 mm. Thus, for a short surface crack, it is essential to select the ultrasonic transducer with higher frequency. For a long surface crack, ultrasonic transducer with lower frequency can provide better result.

The signals shown in Figures 2, 4, 5 and 9 are completely separated in time domain. This was done for clarity. They may overlap as shown in Figures 10 and 11. In such cases, it is not easy to evaluate the time delay between two overlap signals in time domain. The frequency spectrum analysis proposed in this study is capable of extracting the time delay between two overlap signals. In this work, the frequency spectrum analysis is employed to extract the time delay between two signals. In addition, the frequency spectrum can be used to determine the attenuation of signals as a function of frequency and phase shift of one signal to the other in a single test [34]. For ultrasonic technique, these indicate the capability of measuring the crack length, thickness, acoustic impedance and detecting the debonds. The novelty of the frequency spectrum analysis is that it can be applied to many practical problems as mentioned above.

## 5. Conclusions

The depth of surface crack in a solid is examined using ultrasonic technique. The ultrasonic sizing procedure presented in this study combines time domain and frequency spectrum to detect the crack depth. The pulses reflected from both the tip and mouth of the surface crack were transformed to frequency domain using Fourier transform. The time delay between the two pulses is deduced from the frequency spectrum. Reasonable accuracies were achieved with measurement errors less than 7%. The effect of longitudinal wave frequency on the sizing detection is investigated. Experimental test results show that for a short surface crack, it is helpful to select the ultrasonic transducer with a higher frequency. For a long surface crack, a lower frequency can achieve a better result.

## Figures and Tables

**Table 1.** Ultrasonic measurement results of the crack depth using three different frequencies.

**Crack Depth**		**1 mm**		**1.5 mm**		**2 mm**		**3 mm**	

		**Result**	**Error %**	**Result**	**Error %**	**Result**	**Error %**	**Result**	**Error %**
Frequency	2.25 MHz	*	*	*	*	*	*	2.79	7.0
	5 MHz	0.99	1.0	1.49	0.7	1.94	3.0	2.97	1.0
	10 MHz	0.95	5.0	1.43	4.7	1.90	5.0	2.85	5.0
**Crack Depth**		**4 mm**		**6 mm**		**8 mm**			

		**Result**	**Error %**	**Result**	**Error %**	**Result**	**Error %**		

Frequency	2.25 MHz	3.71	7.3	5.57	7.2	7.43	7.1		
	5 MHz	4.05	1.3	6.37	6.2	*	*		
	10 MHz	3.80	5.0	*	*	*	*		

* denotes the crack depth cannot be determined.
